# Visual threats reduce blood-feeding and trigger escape responses in *Aedes aegypti* mosquitoes

**DOI:** 10.1038/s41598-022-25461-2

**Published:** 2022-12-09

**Authors:** Nicole E. Wynne, Karthikeyan Chandrasegaran, Lauren Fryzlewicz, Clément Vinauger

**Affiliations:** 1 Department of Biochemistry, Virginia Polytechnic Institute and State University, Blacksburg, VA 24061 USA; 2 Center for Emerging Zoonotic and Arthropod-Borne Pathogens, Virginia Tech, Blacksburg, VA 24061 USA

**Keywords:** Behavioural ecology, Sensory processing, Motion detection, Animal behaviour, Entomology

## Abstract

The diurnal mosquitoes *Aedes aegypti* are vectors of several arboviruses, including dengue, yellow fever, and Zika viruses. To find a host to feed on, they rely on the sophisticated integration of olfactory, visual, thermal, and gustatory cues emitted by the hosts. If detected by their target, this latter may display defensive behaviors that mosquitoes need to be able to detect and escape in order to survive. In humans, a typical response is a swat of the hand, which generates both mechanical and visual perturbations aimed at a mosquito. Here, we used programmable visual displays to generate expanding objects sharing characteristics with the visual component of an approaching hand and quantified the behavioral response of female mosquitoes. Results show that *Ae. aegypti* is capable of using visual information to decide whether to feed on an artificial host mimic. Stimulations delivered in a LED flight arena further reveal that landed *Ae. aegypti* females display a stereotypical escape strategy by taking off at an angle that is a function of the direction of stimulus introduction. Altogether, this study demonstrates that mosquitoes landed on a host mimic can use isolated visual cues to detect and avoid a potential threat.

## Introduction

Mosquitoes are responsible for transmitting disease-causing pathogens that kill more than 700,000 people every year^1^. The underlying motivation for adult females of epidemiologically problematic mosquito species is their need for nutrients, including proteins, contained in vertebrate blood to produce progeny. In addition to physiological stresses associated with the ingestion of blood, accessing a resource that is hidden under the skin of mobile and defensive hosts is not free of any risks. Hosts display defensive behaviors aimed at deterring or killing mosquitoes^2^. The reproductive fitness of female mosquitoes is thus not only directly linked to their ability to detect, locate and feed on a host but also to survive interactions with a larger and potentially defensive organism.

In humans, a typical response to biting insects is swatting. From the insect’s perspective, a swat corresponds to a rapidly approaching appendage on an interception course, most often a hand. When approaching, the appendage induces rapid air displacement (i.e., mechanical component) and the rapid expansion of an object in the mosquito visual field (i.e., visual component). Previous studies investigating the role of visual cues in triggering escape behaviors in lizards^3^, frogs^4^, mice^5^, larval zebrafish^6^, crabs^7,8^, locusts^9,10^, cricket^11^, sand flies^12^ employed variations of looming stimuli, some designed to closely mimic their main predator while others were designed with specific emphasis on characteristics of the looming object itself, such as its angular velocity and direction^13,14^. The results from these studies found that the likelihood of a prey’s escape response was contingent on specific characteristics of the looming stimuli. For example, fiddler crabs rely heavily on the retinal speed of the stimulus to trigger a “home run” or a full escape where they sprint back to their burrow^15^. In fruit flies, dropping a physical black disk onto a landed fly revealed that escape take-offs rely on a different neuroethological pathway than voluntary, spontaneous take-offs, where the insect pushes on its legs to increase the speed of its response^16^. High-speed videography further revealed that these jumps, mediated by giant fiber neurons that link brain visual areas to wing muscles^13,17–19^, pushed flies directly away from the looming stimulus^20^.

Using a mammalian tail simulator, Matherne et al.^21^, showed that the combined visual cues and airflow generated by the swinging of mammals’ tails reduced the proportion of *Aedes aegypti* landing by 50%. In free flight, both *Ae. aegypti* and the nocturnal malaria vector *Anopheles coluzzii*, displayed rapid escape maneuvers when stimulated with an artificial swatter that induces both airflow and visual cues^22,23^, hinting at the role of visual cues in signaling threats. Furthermore, some mosquito species display a “pre-biting” rest^24^ or “pre-attack” resting^25^ behavior when the mosquito density increases around a target host^26^, which is known to heighten host defensive behaviors^27,28^. With an increase in host defenses but ultimately an endless supply of blood, it would appear adaptive for the mosquito to wait until the host activity calms. However, what sensory cues mosquitoes use to gauge their hosts’ defensiveness remains unclear.

Here, we hypothesize that vision contributes to mediating mosquito escape behaviors. To test this hypothesis, we describe an experimental paradigm geared towards characterizing mosquitoes' responses to isolated visual threats (i.e., fully decoupled from mechanical cues). Specifically, to investigate the escape responses of *Ae. aegypti* mosquitoes landed on a host in response to a visually perceived threat, we simulated a predator-like looming (expanding) stimulus that mimicked the visual properties of a slapping hand since humans are *Ae. aegypti’s* preferred host^29,30^. In a first series of experiments, we used artificial blood-feeding assays to quantify visual threats' effects on mosquitoes' propensity to land and feed on a host mimic. Next, we relied on free-flight assays to define the response characteristics of visually-induced escape responses.

## Results

### Extracting visual features of a human swat

**Figure 1:**
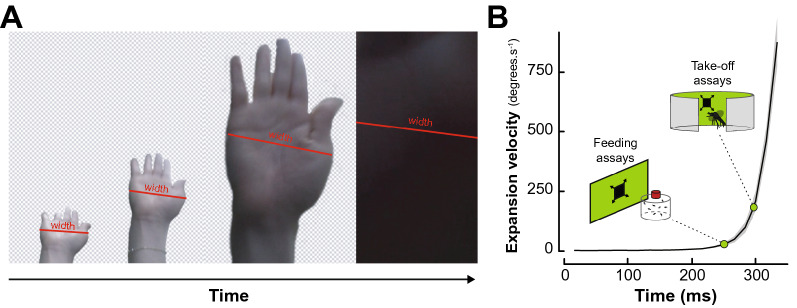
Developing an artificial visual swat. **(A)** Representative screenshots from the video recording of hand slaps filmed at 60 frames per second. The red lines illustrate the measurement of the widest part of the hand. Front view recordings were paired with side-view recordings used to quantify the linear velocity of the hand (data not shown). **(B)** Mean expansion velocity (in degrees per second) of a hand swat as a function of time (black line; n = 10 replicates). Grey shaded area indicates the standard error to the mean expansion velocity. Green points indicate the expansion velocities used to program the expanding squares displayed in the feeding and take-off assays, respectively.

To present ecologically relevant stimuli, we video-tracked a slapping hand on a collision course with a fictive mosquito located at the camera’s position (GoPro Hero 6 in “linear” recording mode at 60 fps, GoPro, San Mateo, CA, USA). Video tracking the hand’s width at the widest point below knuckle height showed an average duration of 335 ± 82 ms with an exponentially increasing expansion rate peaking at 874.9°.s^−1^ before covering the entire field of view of the camera (n = 10; Fig. 1).

**Figure 2:**
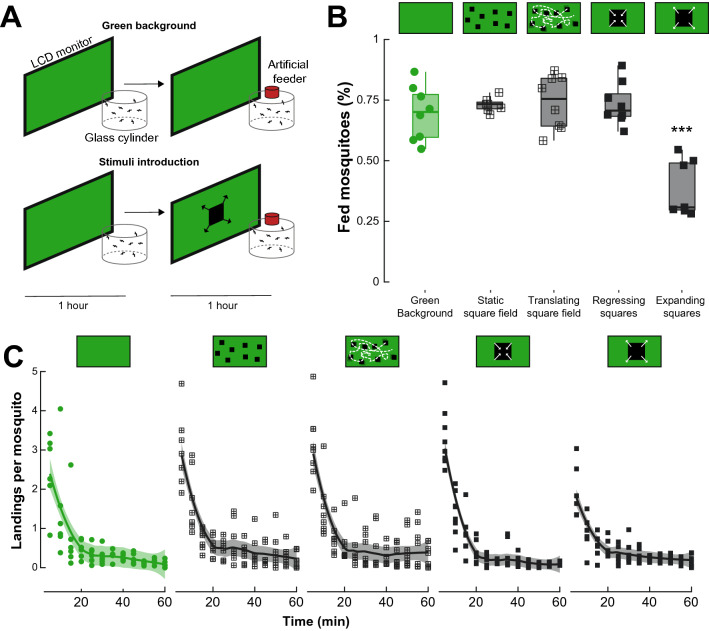
Landing and feeding on a host mimic in different visual contexts. **(A)** Schematic of the experimental apparatus. Groups of mosquitoes are enclosed in a glass cylinder positioned in front of an LCD monitor. To acclimate to the experimental conditions, mosquitoes are left unperturbed in front of a green background for 1 h. Control groups (top) were exposed to a second hour of green background during which the number of landing and feedings on a membrane feeder were quantified. Treatment groups (bottom) were exposed to randomly positioned squares following the patterns described in (**B,C**). **(B)** Proportion of feedings quantified at the end of the second hour of the experiment for the control (green, n = 8) and treatment groups (dark grey, crossed squares for square fields, and filled squares for regressing and expanding squares, 7 < n < 8). **(C)** Number of landings per mosquito quantified every five minutes of the second hour of the experiment for the control (green) and treatment groups (dark grey, crossed squares for square fields, and filled squares for regressing and expanding squares). Asterisks denote significant differences (***p < 0.001; generalized linear model).

**Figure 3:**
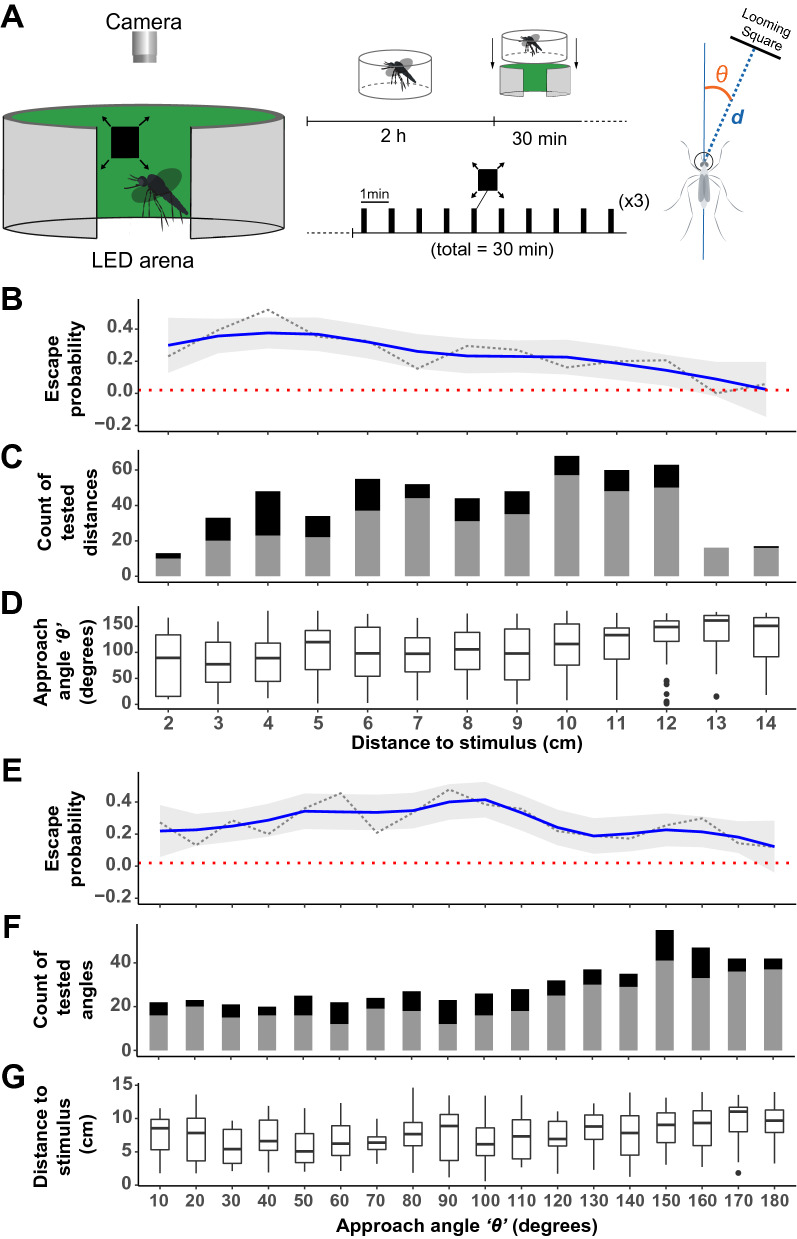
Escape probability as a function of the distance and direction of the stimulus.** (A)** Schematic of the experimental apparatus (left) and graphical representation of the experiment’s timeline (middle) where an individual female mosquito is isolated in a clear cylinder 2 h before the start of the experiments. The cylinder is positioned in the LED arena and the mosquito is provided 30 min to acclimatize to the conditions before being stimulated with a looming square every minute for 30 min. For each stimulus introduction, the angle of approach *θ* and the distance between the head of the mosquito and the center stimulus were measured (Right). **(B,E)** Mosquitoes’ probability to escape (take-off) after introduction of the expanding stimuli, represented as a function of the distance to the point of stimulus introduction **(B)** or as a function of the direction of stimulus introduction, i.e.*,* approach angle **(E)**. A trendline (solid, blue) from a single-term linear model fit with 95% confidence interval (gray) summarizes the relationship between escape probability (doted, black) and the predictor variables. The dotted horizontal line (red) denotes the baseline escape probability (take-off) in the absence of any expanding stimuli. **(C,F)** Number of experimental trials categorized based on mosquitoes response (black) and lack of response (gray) after introduction of the expanding stimuli. **(D)** Distribution of the approach angle θ (degrees) i.e.*,* angle at which the stimulus is introduced relative to the mosquito’s body orientation, across distance bins. **(G)** Distribution of the mosquitoes’ distance from the point of stimulus introduction (cm) across bins of approach angles. In **(B–D)**, the response variables are visualized as a function of the mosquitoes’ distance from the point of stimulus introduction (0–14.63 cm) represented as a categorical variable, i.e.*,* 1 cm per category. In **(E–G)**, the response variables are visualized as a function of the approach angle θ (0°–180°, where 0° is to the front and 180° is behind the mosquito) represented as a categorical variable, i.e.*,* 10° per category.

Side view recordings of ten slaps, showed that the peak instantaneous velocity of the slap was of 8.01 ± 0.66 m.s^−1^, which is slower, but within the same order of magnitude as the recorded speed of Olympic boxers’ punches (i.e., ~ 9.14 ± 2 m.s^−1^^31^). The duration of the swat was used to program a dark looming square displayed to the mosquitoes on either an LCD monitor or on a programmable LED arena (Figs. 2A,3A, respectively). The expansion velocity of the fictive swats used throughout the experiment was kept constant at 28.9 horizontal degrees.s^−1^ (LCD monitor) and 183.6 horizontal degrees.s^−1^ (LED arena) as seen from the center of the mosquito container. These virtual swats correspond to the expansion velocity of a hand at 80 ms and 40 ms before contact, respectively. The rationale behind this choice was to simulate earlier phases of the swat in the feeding assays and later moments (i.e., closer to impact) in the take-off experiments, as the distance from the display was greater in the feeding assays than in the take-off experiments (300 mm versus 118.5 mm, respectively).

### Visual motion reduces feeding, but not landing, on a warm source of blood

To analyze whether visual, threat-like stimuli could impact mosquitoes’ ability to blood-feed, groups of adult females were provided access to a host mimic positioned in front of a visual display (Fig. 2A). On average, 69.6 ± 3.97% of mosquitoes fed to repletion in front of the green background (Fig. 2B). Similarly, 73.1 ± 1.11% of mosquitoes fed in front of a field of static small squares, 74.1 ± 3.93% in front of translating small squares, and 73.4 ± 3.14% in front of a single repeatedly regressing square (Generalized linear model: *p* > 0.356; Fig. 2B). However, this proportion was significantly reduced when expanding squares were introduced at regular intervals (38.7 ± 4.38%; Generalized linear model: *p* < 0.001; Fig. 2B).

In the presence of a uniform green background (negative control), an average of 204 ± 25.2 total landings on the feeder were observed over the course of each trial (n = 8; 1226 total landings). When multiple static squares were randomly displayed on the screen, 277 ± 32.2 landings per trial were counted (n = 7; 1940 total landings), comparable to 280 ± 48.6 landings when randomly positioned squares followed a translational “random walk” on the screen (n = 8; 2240 total landings). When repeatedly presented with a single regressing square 212 ± 18.1 landings were counted per trial (n = 8; 1695 total landings), but only 174 ± 17.8 landings per trial were observed when expanding squares were repeatedly presented to the mosquitoes (n = 7, 1042 total landings). Although there was a tendency for, overall, fewer mosquitoes to land when exposed to looming squares, this effect was only marginally significant (Generalized linear model: *p* = 0.0514; Fig. 2C). Furthermore, while significantly more mosquitoes landed during the early phases of the experiments (Generalized linear model: *p* < 0.032; Fig. 2C), the difference between stimuli was not significant (Generalized linear model: *p* > 0.288; Fig. 2C).

### Mosquitoes’ response to visual threats is a function of the distance and direction of the stimulus

To better understand the role of looming visual objects in the context of threat avoidance, mosquitoes were stimulated at regular intervals, with the expanding stimulus being introduced at random positions around an LED-based arena (sensu^32^) consisting of an array of 96 × 16 LEDs subtending 360° horizontally and 54° vertically from the center of the arena. Because mosquitoes were free to fly (or not), this randomization led to a large number of randomized directions of stimulus introduction relative to the mosquito, and random distances between the location of the mosquito and the point of stimulus introduction on the arena (Fig. 3B–G). Mosquitoes’ responses to expanding stimuli introduced at all directions were sampled homogeneously, with stimulations right behind the mosquito (> 140°) being sampled relatively more than the rest (Supplementary Table 1). Likewise, the mosquitoes’ responses as a function of their distance to the expanding stimuli were sampled homogeneously except for positions where the mosquitoes were either too close (< 3 cm in distance) to or too far (> 12 cm in distance) from the stimuli (Supplementary Table 2).

In the absence of any stimulation, the probability of take-offs was 0.02 (dotted red line; Fig. 3B,E). This number significantly increased to 0.26 when an expanding stimulus was introduced (including stimuli delivered from all directions and distances; Binomial Exact test: *p* < 0.001). However, mosquitoes’ escape probability varied significantly as a function of their distance to the stimulus, with peaks for stimuli delivered between 3 and 4 cm away from the mosquito (escape probability = 0.52; Generalized linear model, *p* < 0.001). It is worth noting that the lower escape probability observed for the closest distance bin (2 cm) could be due to the lower sampling of this distance bin. While the effect of the angle at which the expanding square was introduced was not significant (Generalized linear model, *p* = 0.056), the probability of escape tended to peak at angles between 60° and 90°, i.e.*,* laterally to the mosquito.

**Figure 4:**
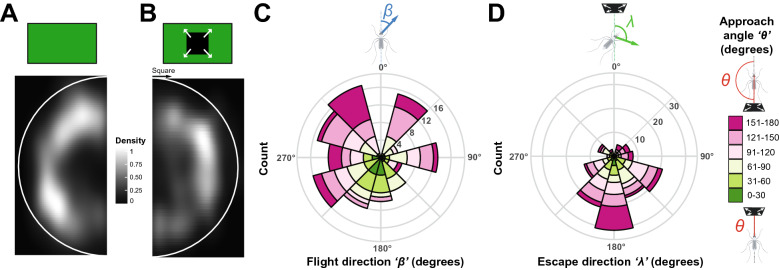
Overall behavioral responses to the looming square. **(A,B)** 2-D histogram of the locations occupied by mosquitoes within the LED arena. Data are mirrored on the vertical axis; the white semi-circles indicate the relative position of the arena’s walls. **(A)** Normalized histogram of the location of mosquitoes during randomly selected flight bouts, without visual stimulation. **(B)** Normalized histogram of the location of mosquitoes during the first 0.17 s (5 frames) after a take-off triggered by the introduction of a looming square. Data are oriented such that the stimulus introduction (indicated with a black arrow) is positioned at the top of the figure. **(C)** Rose diagram showing the circular distribution of the flight direction, *β* (i.e.*,* the direction of escape relative to the mosquito’s body orientation) within 0.17 s of stimulus-triggered take-offs. **(D)** Rose diagram showing the circular distribution of escape direction, *λ* (i.e.*,* the direction of escape relative to the stimulus) within 0.17 s of stimulus-triggered take-offs. (**C,D**) The length of the bars encodes for the number of observations, color-coded as a function of the approach angle, θ where 0° is to the front of the mosquito, and 180° is behind the mosquito (n = 142).

To confirm that mosquitoes were indeed evading the expanding square, we compared the spatial occupancy of the arena between mosquitoes that were in flight but not stimulated and mosquitoes that took off following stimulus introduction. Because of the randomization of the stimulus introduction, data were rotated to align all stimuli introductions at 0°. While non-stimulated mosquitoes avoided the center of the arena, they explored the entire periphery of the device (Kuiper’s one sample test of uniformity: n.s.; test statistic = 1.6443, critical value at α = 0.05: 1.747; Fig. 4A). On the other hand, stimulated mosquitoes not only avoided the center of the arena, but also flew to the sectors opposed to where the stimulus was introduced, and avoided the fictive line of interception with the virtual stimulus (Kuiper’s one sample test of uniformity: p < 0.05; test statistic = 1.8243 critical value at α = 0.05: 1.747; Fig. 4B).

To further characterize the escape response displayed by mosquitoes, we analyzed the ‘flight direction’ (denoted as *β*, i.e.*,* the take-off direction relative to the mosquito’s body orientation) and ‘escape direction’ (denoted as *λ*, i.e.*,* the direction of escape relative to the stimulus) as a function of both the ‘approach angle’ (denoted as *θ*, i.e.*,* the angle at which the stimulus is introduced relative to the mosquito’s body orientation) and the distance to the stimulus (Supplementary Figs. S4, S5).

Regardless of the approach angle and distance to the stimulus, mosquitoes did not exhibit a preferred flight direction relative to their body orientation (Rao’s Spacing Test of Uniformity; *p* > 0.05; Fig. 4C). However, responding mosquitoes significantly escaped away from the direction of the stimulus (Rao’s Spacing Test of Uniformity; *p* < 0.05; Fig. 4D, Supplementary Figs. S3, S4, S5). This is explained by the significant effect of the angle of approach on the direction of escape (Generalized linear model; *p* < 0.001), where mosquitoes predominantly escaped by flying towards their back when the stimulus was introduced in front of them, i.e., moving away from the direction of the stimulus (closer to 0°, green bars; Fig. 4C, Supplementary Video 3). Mosquitoes that had stimuli introduced laterally (around 61°–150°) escaped by performing a slight turn relative to the direction of the stimulus, and mosquitoes that had stimuli introduced behind them (< 150°) escaped by flying forward, i.e., orienting the most away from the origin of the stimuli (Fig. 4D; Supplementary Figs. S4, S5). Although there was a tendency for mosquitoes to go further away when the stimulus was introduced closer to them (blue vectors in Supplementary Fig. S5A), the distance to the point of stimulus introduction had no significant effect on the displacement after take-off, regardless of the approach angle *θ* at which the stimulus was introduced relative to the mosquito’s body orientation (Supplementary Fig. S5B).

**Figure 5:**
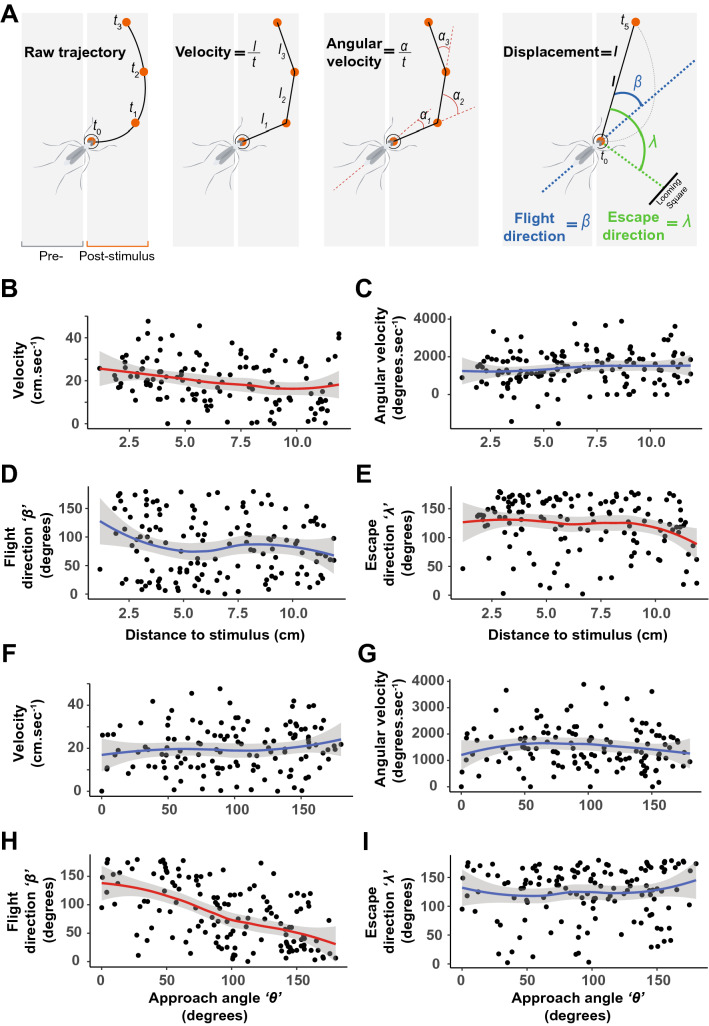
Statistical analysis of the escape strategy displayed by mosquitoes. **(A)** Illustrations of the mosquitoes’ responses quantified as **(B,F)** velocity (cm.s^−1^), **(C,G)** angular velocity (degrees.s^−1^), **(D,H)** flight direction, *β* (degrees) i.e.*,* the direction of escape relative to the mosquito’s body orientation, **(E,I)** escape direction, *λ* (degrees) i.e.*,* the direction of escape relative to the stimulus, and displacement (see Supplementary Fig. S5). In **(B–E)**, the response variables are visualized as a function of the mosquitoes’ distance to the stimulus (0–11.89 cm). In **(F–I)**, the response variables are visualized as a function of the ‘approach angle’ *θ*, where 0° is in front of the mosquito and 180° is behind the mosquito. In (**D,H**), a *β* angle of 0° indicates a forward flying mosquito and 180° indicates a backward flying mosquito. In **(B–I)**, a trend line from a single-term local regression model fit with 95% confidence interval (gray shaded area) summarizes the relationship between the response and predictor variables. Red trend lines indicate significant effects of the predictive factors (distance to stimulus or angle of stimulus introduction, respectively) on the response variables, and blue trend lines indicate non-significant effects (generalized linear models, *α* = 0.05).

Analyzing four aspects of the escape response (i.e.*,* velocity, angular velocity, flight direction, and escape direction; Fig. 5A) we found that the velocity and escape direction (*λ*) were significantly influenced by the distance to the point of stimulus introduction (Generalized linear models: *p* = 0.003, *p* = 0.138, *p* = 0.265, and *p* = 0.042 for the velocity, angular velocity, beta, and lambda, respectively; Fig. 5B–E). When analyzed as a function of the angle of stimulus introduction, only the flight direction (*β*) was significantly affected (Generalized linear models: *p* = 0.374, *p* = 0.851, *p* < 0.001 and *p* = 0.401 for the velocity, angular velocity, *β*, and *λ*, respectively; Fig. 5F–I). In Fig. 5D,H, a *β* angle of 0° indicates a forward flying mosquito and 180° indicates a backward flying mosquito. In Fig. 5E,I, a *λ* of 0° indicate flying toward the stimulus and 180° away from the stimulus. These results show that mosquitoes escaped away from the looming stimulus, regardless of the approach angle, by flying away from the direction of the stimulus.

**Figure 6:**
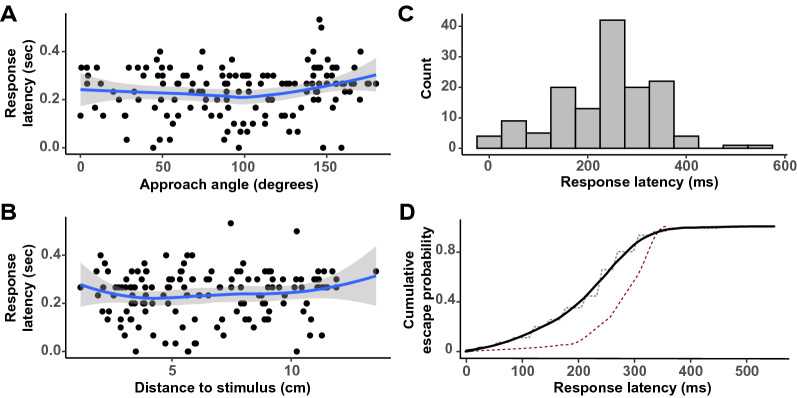
Response latency. Latency associated with the take-off responses of mosquitoes (s) stimulated with a looming square visualized as a function of the **(A)** approach angle, *θ* where 0° is to the front of the mosquito and 180° is behind the mosquito **(B)** distance to the stimulus’ point of introduction (cm). **(C)** Histogram visualizing the distribution of response latency associated with the take-off responses of 142 mosquitoes. **(D)** The cumulative probability of take-off in relation to the time since stimulus introduction (black line: smoothed using a local polynomial regression *loess*; dashed grey line: raw probability calculated in 10 ms bin increments). For comparison, the dashed red line indicates the cumulative probability of take-off in *Drosophila melanogaster* (adapted from^20^).

Remarkably, take-offs were even observed even for stimulus introductions immediately behind the mosquito (135°–180° angles), highlighting landed mosquitoes’ capacity to detect threats coming from all directions along the azimuth. Finally, analyzing the response latency of mosquitoes, we found that the time taken by the mosquitoes to take off in response to stimulus introduction was not influenced by the approach angle *θ* of the stimulus and mosquitoes’ distance to the stimulus. The response latency of the 142 escape responses observed in this study typically ranged between 0 and 0.53 s and was non-normally distributed (Fig. 6; Shapiro–Wilk test; *p* < 0.001). The cumulative probability of take-off started to plateau at around 350 ms, which is similar to previous observations in *D. melanogaster*^20^, although the probability tended to ramp up earlier in mosquitoes (Fig. 6D).

## Discussion

Our results demonstrate that landed mosquitoes can use isolated visual cues to detect and respond to potential threats, i.e., even in the absence of other sensory information (*e.g.*, air displacement due to the host’s movements). In our feeding assays, we found that the presence of looming visual cues did not impact the number of landings on the artificial feeder. However, the proportion of mosquitoes fed to repletion was reduced by nearly 50%. Interestingly, other types of visual motion (i.e., translation, regression) did not impact landing and feeding proportions (Fig. 2). This indicates that while the presence of rapidly moving objects in the visual field of flying mosquitoes does not impair their ability to navigate toward a source of food, once landed, mosquitoes specifically respond to looming visual objects by displaying an escape response (Fig. 2B). Although the identity of each mosquito was not maintained in our analysis, the large number of landings relative to the number of feeding events suggests that the same mosquitoes made several landing attempts and that several mosquitoes landed (several times) but did not feed.

Using an LED-based flight arena, we were able to further characterize this response in landed mosquitoes by introducing expanding squares at randomized distances and directions relative to the focal mosquito. Results confirmed that looming squares trigger mosquitoes to take off at a higher probability than chance (i.e., compared to the take-off probability of landed mosquitoes randomly sampled in the absence of visual stimulation: 0.02). The peak escape probability (0.52) observed here was comparable to the frequency of successful escapes from a mechanical swatter by in-flight *Ae. aegypti* females^22,33^. The closer the mosquito to the stimulus, the more likely it was to take off, and although all directions of introduction induced take-offs, threats originating laterally tended to elicit more escapes (Fig. 3). In particular, mosquitoes were most responsive to looming stimuli that came from approximately 60°, 90°, and 100°. While the heterogeneity of the mosquito retina^34^ has been described, it is too early to conclude about the spatial specialization for detecting threats. Further studies will be required to determine the range of elevations above the plane of the substrate at which mosquitoes can detect threats, especially because the field of view available to a mosquito actively engaged in blood-feeding may differ from its resting position (*e.g.*, different head angle, substrate orientation). Such a spatial specialization has, however, been found in tethered *Drosophila melanogaster*, where the expansion of centrally versus laterally positioned objects elicited different response profiles: frontal objects induced strong leg and wing-beat frequency responses but minimal changes in wing-beat amplitudes while lateral objects elicited stronger changes in wing-beat amplitude and transient increases in wing-beat frequency, but did not evoke leg responses^35,36^.

Remarkably, the escape responses we observed were not random: mosquitoes rapidly positioned themselves out of the line of interception with the trajectory of the fictive threat (Fig. 4B). Analysis of the trajectories displayed after take-off shows that the flight direction is indeed directly influenced by the angle of stimulus introduction (Figs. 4,5H), which allows mosquitoes to follow an escape direction oriented away from the point of introduction of the stimulus (Figs. 4C, 5I). A similar behavior had been observed in flight in *D. melanogaster* where looming targets triggered visually-directed banked turns that reoriented the fly’s path within a handful of wingbeats^12^. In addition, studies focusing on the take-off behavior of *D. melanogaster* showed the same relationship between the approach angle of a visual threat and the direction of the jump flies performed as the first step of their escape strategy^20^. It is worth noting that in flies, *D. melanogaster* displays two types of take-off strategies as a function of the context (i.e., a long mode and short mode take-off) while in mosquitoes only one strategy has been observed during voluntary and escape take-offs^16,18,37^. Specifically, the analysis of the take-off behavior of blood-fed *An. coluzzii* showed that mosquitoes reach comparable take-off speeds as *D. melanogaster*, but with a fraction of the push-off forces^37,38^. This suggests that mosquitoes may have adapted to escaping their hosts without alerting them, but since no visual threats were introduced in these studies, a fine-scale analysis of landed mosquitoes escaping a visual threat will be required to determine whether they adjust their take-off strategy when threatened. In particular, we observed that the closer the mosquito was to the point of introduction of the threat, the faster it escaped (Fig. 5B). Although the distance to the stimulus and its angle of approach were randomized, it is worth noting that the cumulative probability of escape we observed was overall similar to what has been previously described in fruit flies (Fig. 6D). Future studies providing direct comparisons between flies and mosquitoes will be required for a truly quantitative assessment of mosquitoes’ adaptations to feeding on a defensive host.

It is also possible that the response probability to the looming stimuli was actually higher than what we observed by only quantifying take-offs, as responses may have taken other forms, such as a freeze response to threats. Indeed, other animals have been shown to make a decision to either freeze or flee when a threat is presented^5^. Given that freezing responses could not be identified and characterized, we focused on landed mosquitoes to analyze the fleeing responses. In addition, because of the size of the clear acrylic cage the mosquitoes were contained in, escape responses could only be examined over a short period of time, as mosquitoes would rapidly reach the wall of the container and redirect their flight trajectory (Supplementary Video 3). However, despite this limitation and while quantifying responses in 2-dimensions only, our results show clear visual responses to isolated visual stimuli: similarly to flies^16,20^, mosquitoes displayed a tight relationship between the angle at which the stimulus is introduced and the flight direction. Within 1/6th of a second, mosquitoes did not just randomly scatter. Instead, they flew away at an angle directly influenced by the approach angle of the stimulus to escape on a trajectory mostly away and at a slight angle from the stimulus (Fig. 5H,I).

Altogether, results from the present study provide some insights into the “pre-biting’ or “pre-attack” resting behavior previously observed in several anopheline and culicine mosquito species^26^. Because this “pre-biting” rest was more frequently observed at higher mosquito densities, one could hypothesize that mosquitoes possessed the ability to evaluate the level of defensiveness from afar, possibly using vision in this context. In this hypothetical scenario, mosquitoes would land near the host and wait for its defensive behaviors to decrease before approaching, thus increasing the probability of successful blood-feeding. However, in our artificial feeding assays, the number of landings on a host mimic was not affected by visual threats, only the proportion of successful feedings on an otherwise defenseless feeder. This suggests that the pre-biting behavior would require mosquitoes to land on the host and experience its defensive behavior, which differs from other studies conducted on tabanid flies, where stripes present on the fur of the host made it much more difficult for the flies to land on them^39^. In this case, visual cues directly impaired the insects’ landing ability, and, as a result, horses with solid color experienced more fly landings.

In nature, as mentioned above, a critical contributor to mosquitoes’ feeding success is the density of female mosquitoes around the host^27,28,40,41^. Indeed, as the density of mosquitoes increases, so does the level of defensiveness of the host, which negatively correlates with the proportion of females that successfully blood-feed^28^. Consequently, mosquitoes prefer to feed on the least defensive or least active host over more defensive ones^40,42^. While our results suggest that *Ae. aegypti* gauges the defensiveness of a host only after landing on it, it is worth highlighting that only visual cues were available in our experiments. But while mechanical cues (i.e.*,* air displacement) generated by a mammal tail simulator significantly prevented mosquitoes from landing^21^, whether they can trigger the pre-biting resting behavior remains to be determined. In a previous study, when a mechanical vibration calibrated to mimic an average swat was repeatedly paired with host olfactory cues, *Ae. aegypti* females learned the association between the mechanical stimulus and the odor and subsequently avoided the trained odor^43^. Mechanical cues thus contribute to host selection processes^2,43,44^ and, by disturbing the ability of mosquitoes to land on the host, most likely contribute to a mosquito’s evaluation of the host’s defensiveness. This relationship between mosquito density, host defensiveness, and feeding success is central to the vectorial capacity of mosquitoes, and the results of the present study suggest that visual cues may be used by landed mosquitoes to gauge the defensiveness of a host.

Beyond their role when presented in isolation, sensory cues from multiple modalities (e.g*.*, vision and olfaction) are integrated by the mosquito brain^45–47^. In the context of evading host defenses, Cribellier et al.^22^ showed that, in flight, *Ae. aegypti* females were most successful at evading a mechanical swatter in bright light conditions, i.e., when both visual and mechanical cues were available. Interestingly, night-active *An. coluzzii* were most successful at escaping in the dark, suggesting that nocturnal mosquitoes maximize their escape performance by adjusting the parameters of their flight behavior itself, such as adopting a more stochastic (i.e., protean) flight^33^.

In the present study, we provide an experimental paradigm that permits the presentation of visual cues in isolation from mechanical perturbations. Our Results show that visual threat-like stimuli alone are sufficient to disrupt *Ae. aegypti*’s blood-feeding behavior and trigger a stereotypical escape response in landed females, influenced by the distance and direction of the threat. This work opens new research avenues for improving our understanding of the role of vision in mosquito biology and developing control strategies that target this sensory modality. For example, the experimental paradigm presented here could be adapted to spatially (and temporarily) decouple visual and mechanosensory cues to deepen our understanding of multimodal sensory integration processes in the context of threat avoidance.

## Methods

### Mosquitoes

Wild type *Aedes aegypti* mosquitoes (Rockefeller strain, MR-734, MR4, ATCC®, Manassas, VA, USA) were used throughout the experiments. The colony was maintained in a climatic chamber set at 25 ± 1 °C, 60 ± 10% relative humidity (RH), and under a 12-12 h light–dark cycle. Cages of adults were fed weekly using an artificial feeder (D.E. Lillie Glassblowers, Atlanta, Georgia; 2.5 cm internal diameter) with heparinized bovine blood (Lampire Biological Laboratories, Pipersville, PA, USA) heated at 37 °C using a circulating water-bath. Between blood-meals, mosquitoes were fed ad libitum with 10% sucrose. No vertebrate hosts were used to rear mosquitoes or during the experiments. Eggs were collected from blood-fed females and hatched in deionized water. Larvae were reared in groups of 200 in covered pans (26 × 35x4 cm) containing deionized water and fed ad libitum with fish food (Hikari Tropic 382 First Bites—Petco, San Diego, CA, USA). Pupae, in groups of 100, were isolated in 16 oz containers (Mosquito Breeder Jar, Bioquip Products, Rancho Dominguez, CA, USA) until emergence.

For all the experiments, 6–8 days old female mosquitoes were used. They were kept in the presence of males, fed ad libitum with 10% sucrose until 24 h prior to the experiments, and never blood-fed. This gave mosquitoes the time to mate in the containers before the experiments (dissection of randomly selected females revealed that 95% of them had oocytes) and ensured high motivation to seek hosts for blood feeding^48,49^. All experiments were performed during the last four hours of the mosquitoes’ subjective day, i.e., *Zeitgeber Time* (ZT) 8–12 ^50,51^.

### Expanding stimuli

Tracking of the hand’s width of the first author over ten replicates provided an average duration and expansion velocity profile of a slap (ImageJ; National Institutes of Health, USA; Fig. 1). This information was used to program the symmetrical expansion of a dark square over a bright green background. A square was selected over other shapes as behavioral responses to expanding squares have been shown in tethered *Ae. aegypti females*^47^, and the response characteristics of classical models such as *Drosophila melanogaster* have been extensively analyzed^36^. Furthermore, in the programmable LED arena, a square allowed for the expansion of the stimulus along both dimensions with a higher step resolution than, for example, a disk.

### Artificial blood-feeding assays

#### Procedure

Visual patterns generated with PsychoPy v3.0 ^52^ were displayed on a 29-in. (73 cm diagonal) LED-backlit LCD monitor (UltraSharp 29 Ultrawide Monitor—U2917W, Dell, Round Rock, TX, USA). Specifically, five patterns were generated:

1. A uniform green screen (negative control).
2. A “square field” composed of fifty static squares randomly positioned on the screen, without overlap. Altogether, the 50 squares occupy the same area of the screen as the looming square at its maximum expansion.
3. An animated square field where 50 squares similar to (2) are randomly positioned on the screen and follow a translational motion (i.e., “random walk” without overlap), at a velocity of 5.4 pixels/s.
4. Regressive squares identical to the expanding squares (see 5) in all respect but the direction of motion.
5. Expanding squares programmed to have the same duration as a human swat (Fig. 2A, Supplementary Video 1) and introduced every 0.8 s for a duration of 0.3 s.

The positions of the regressing and looming squares were randomly determined for each experiment trial to account for potential spatial bias. For this purpose, PsychoPy used an array of cartesian coordinates randomized using the *rand* function in Microsoft Excel.

Green, with a peak irradiance at 535 nm, was chosen as a background color to allow comparisons with the conditions in the LED arena (peak irradiance: 571 nm) (Supplementary Fig. S1).

Mosquitoes (21 < n < 35) were isolated into clear glass cylinders (10 × 10 cm), starved from sucrose for 24 h before the experiment, and tested during the last 2 h of the photophase (ZT 10–12). In the first hour of the experiment, the screen was kept uniformly green to allow mosquitoes to acclimate to the experimental conditions. At the onset of the second hour, a membrane blood feeder was placed on the fabric mesh-lined top side of the glass cylinder. The feeder was warmed to 37 ℃ using a circulating water bath thirty minutes prior to the onset of the experiment. The warmed feeder was filled with ~ 5 ml of heparinized bovine whole blood (Lampire Biological Laboratories, Pipersville, PA, USA) fifteen minutes prior to the beginning of the experiment to allow the blood to heat up. The fabric mesh-lined side of the glass cylinder allowed mosquitoes to sense the heat and see the visual contrast of the feeder. The number of mosquitoes that landed and blood-fed on the feeder was recorded using a camera (Logitech C920, Logitech, Lausanne, Switzerland).

In the negative control (2 consecutive hours of green background), the baseline levels of landing and feeding were quantified in front of a uniform green screen. In the first hour, mosquitoes were allowed to acclimatize to the experimental conditions, including the uniform green background. In the second hour, after placing the blood feeder on a glass cylinder enclosure, the number of landings and feedings on the feeder were quantified. In the treatment groups, mosquitoes isolated in the glass cylinder enclosure were acclimatized to the uniform green screen in the first hour, following which the visual stimuli, i.e., either static square field, moving square field, regressing squares, or expanding squares were introduced.

### Statistical analysis

After every trial, the number of engorged females was quantified via visual inspection of their abdomen for the presence of blood, and the number of landings was quantified from the video recordings. These numbers were then compared between treatments as proportions (categorical fixed predictors with two levels: *uniform background* and *looming squares*). For the analysis, we used a Generalized Linear Model assuming a quasibinomial error distribution for the proportion of feeding and a Poisson error distribution for the number of landings per mosquito. The analysis was performed in *R* (version 3.6.2) using the packages *lme4* (version 1.1–27.1^53^) and *multcomp* (version 1.4–17^54^).

### Free-flight LED arena

#### Procedure

Individual mosquitoes were cold anesthetized on ice and separated in clear acrylic cylinders with a clear acrylic lid on the top and a fabric mesh lining at the bottom. These containers were kept at 26 °C for at least two hours to allow the mosquitoes to recover from the cold anesthesia. Containers were next moved to the experimental room (23 ± 1 °C, 45 ± 5% RH) thirty minutes before the start of the experiment to allow the mosquitoes to acclimatize to the ambient conditions. In every experimental trial that lasted for thirty minutes, a container was placed inside the arena, with the expanding stimulus being introduced every minute at a randomized angle around the arena (Fig. 3A, Supplementary Video 2). The distance and the direction to the point of stimulus introduction were a function of both the mosquito’s position in the arena and the randomized angle of introduction. The behavior of mosquitoes was recorded at 30 fps with a camera (Logitech C920, Logitech, Lausanne, Switzerland).

### Video analysis

Mosquitoes that did not move throughout the duration of the experiment (~ 7%) were determined “not active” and discarded from the analysis. Because of the study’s focus on take-off responses, trials in which mosquitoes were in flight before the introduction of the stimulus were discarded from the analysis (n = 63). Based on these criteria, a total of 551 stimuli introductions were conserved, and videos of landed mosquitoes were trimmed 30 s before and 30 s after each stimuli introduction. These one-minute trimmed videos were then converted into image sequences and imported into Fiji ImageJ (National Institutes of Health) for manual continuous tracking of the entire mosquito trajectory using the Manual Tracking plugin. The head of each mosquito was tracked before, during, and after stimulus introduction. The stimuli center points were also tracked for each stimulus introduction. The mosquito-stimulus distance was measured using the measuring tool in Fiji ImageJ and measuring the distance between the center of the stimulus to the mosquito head. The angle of stimulus introduction was measured using the same tool by measuring from the center of the stimulus to the head of the mosquito to the tip of the abdomen. Distances were measured in pixels and the conversions to cm provided throughout the manuscript were obtained by reporting the measured diameter of the arena to its measurement on the videos.

#### Statistical analysis

To calculate mosquitoes’ escape probability, only individuals that took off within 15 frames post-stimulus introduction (i.e.*,* 0.5 s) were considered as “responsive” but, overall, we observed that 98.6% of mosquitoes that responded to the stimulus took off within 0.4 s of the stimulus presentation. Take-offs past the ½ of a second time frame were not considered responses as mosquitoes would have been intercepted by the fictive object beyond that time.

A baseline probability of take-off was determined by repeating the experiments with the exception that no visual looming stimuli were introduced. All LEDs of the arena were kept ON for thirty minutes and the behavior of individualized mosquitoes (n = 10) was recorded with the same video camera as the treatment group. For each mosquito, ten time points were randomly selected within these 30 min, using the sample function in R (i.e., N = 100). At each of these times and for up to five seconds afterward, spontaneous take-offs were quantified and used to calculate the baseline take-off probability in the absence of visual stimuli.

All data were saved as .csv and imported in *R* (version 4.1.3). Trajectories were rotated so that all stimuli introductions were fictively re-positioned at 0°. All the points before the stimulus introduction were considered *pre-stimulus*, while all the points after the stimulus introduction were considered *post-stimulus*. In the *post-stimulus* points, we synchronized the escape responses (N = 142) by discounting mosquitoes’ latency to respond to the expanding stimulus (i.e.*,* trial-to-trial variations in the timing of the take-off response). We opted to analyze the first 5 frames of these synchronized responses as our frame-by-frame analysis revealed that this duration (i.e.*,* 1/6 of a second) effectively captured the escape (take-off) responses of mosquitoes while excluding the effects of mosquitoes hitting the walls of the arena (Supplementary Video 3). From this data, five response variables were calculated by subtracting the *pre-stimulus* period from the *post-stimulus* period (to account for mosquitoes walking before stimulus introduction): linear and angular velocity, flight direction ‘*β*’, escape direction ‘*λ*’, and displacement: (i) the linear velocity was determined by the distance between consecutive points divided by the sampling interval (cm.s^−1^); (ii) the angular velocity was calculated as the change in heading between consecutive points divided by the sampling interval (degrees.s^−1^); (iii) the flight direction ‘*β*’ was calculated as the direction of escape relative to the mosquito’s body orientation (degrees); (iv) the escape direction ‘*λ*’ was calculated as the direction of escape relative to the stimulus and (v) the displacement (distance travelled) was quantified by measuring differences between the mosquito’s location at time of stimulus introduction and its location 5 frames (0.17 s) after the take-off triggered by the introduction of the stimulus. The effects of the distance to stimulus and the approach angle ‘*θ*’ on linear velocity, angular velocity, displacement, and response latency were assessed by means of generalized linear models (GLMs) using the *R* package *lme4* ^53^. The response variables flight direction ‘*β*’ and escape direction ‘*λ*’were analyzed to assess the uniformity and periodicity of circular data using Rao’s spacing test and Rayleigh’s test respectively using the *R* package *circular*^55^.

In order to quantify the amount of digitizing error introduced by manual tracking of the mosquitoes, three experimenters tracked the same 2-s-long sequence (59 frames), once a day during 3 consecutive days. This provided an estimate of the inter- and intra-individual errors in the digitized cartesian coordinates, quantified here as the Root Square Mean Error (RMSE), calculated according to Eq. (1):

1 $$RMSE= \sqrt{{\sum }_{i=1}^{n}\frac{{({x}_{average, i}- {x}_{measured,i})}^{2}+ {({y}_{average,i}- {y}_{measured,i})}^{2}}{n}}.$$

On average, the tracked location of the mosquito deviated by 7.86 pixels (Supplementary Fig. S2) from the mean of the locations measured by all three experimenters. This corresponds to 43.9% of the body length of an average-sized mosquito landed at the bottom of the arena (17.88 ± 0.47 pixels or 4.87 ± 0.12 mm). In this context, and to minimize the influence of errors introduced during the digitization process, changes in velocity and angular velocity were calculated after smoothing the raw data using a low-pass butterworth filter (*pass.filt()*, *R* package *dplR* 1.7.2 ^56^) with a cut-off frequency of 0.25 ^57^. The four response variables thus quantified were visualized as a function of the mosquitoes’ distance from the point of stimulus introduction (Fig. 5B–E) and the angle of stimulus introduction (Fig. 5F–I) with a trendline (± 95% confidence interval) from a single-term local regression model fit summarizing the relationship (*geom_smooth(method* = *‘loess’)*, *R* package *ggplot2* 3.3.5 ^58^).

## Supplementary Information

Supplementary Information.

Supplementary Video 1.

Supplementary Video 2.

Supplementary Video 3.

## Data Availability

The datasets generated during and/or analyzed during the current study are available in the Mendeley Data repository, https://data.mendeley.com/datasets/hhkz8bspsw/1.
