# Supplementary material for: Visual threats reduce blood-feeding and trigger escape responses in Aedes aegypti mosquitoes
Source: Sci Rep. 2022 Dec 9;12:21354. doi: 10.1038/s41598-022-25461-2 (PMC9734121; doi:10.1038/s41598-022-25461-2)
Supplement: Supplementary file 1 — Supplementary Information. [file 41598_2022_25461_MOESM1_ESM.docx]

**Supplementary Information**

**Supplementary Video 1. Illustration of stimuli delivered in the two experiments.**

**Supplementary Video 2. Example of a mosquito taking-off in response to the looming square.**

**Supplementary Video 3. Mosquitoes’ spontaneous flight trajectory differ from escape (take-off) responses triggered by visual stimulation**

Normalized 2-D histograms of the locations occupied by individual mosquitoes (white dots) within the LED arena. The trajectories are rotated to fictively re-position the stimuli introductions at the top at 0°, and data are mirrored on the vertical axis to the left to complement the data visualization in Figure 4A,B. *Left*: positions occupied by mosquitoes during 1 sec (30 frames) of free flight, in the absence of visual stimulation. *Right:* positions occupied by landed mosquitoes ⅓ of a second before introduction of a looming square and ⅔ of a second after stimulation. Stimuli introductions have been fictively repositioned the at the top, at 0°. Introduction of the looming square is time stamped on the video.

**
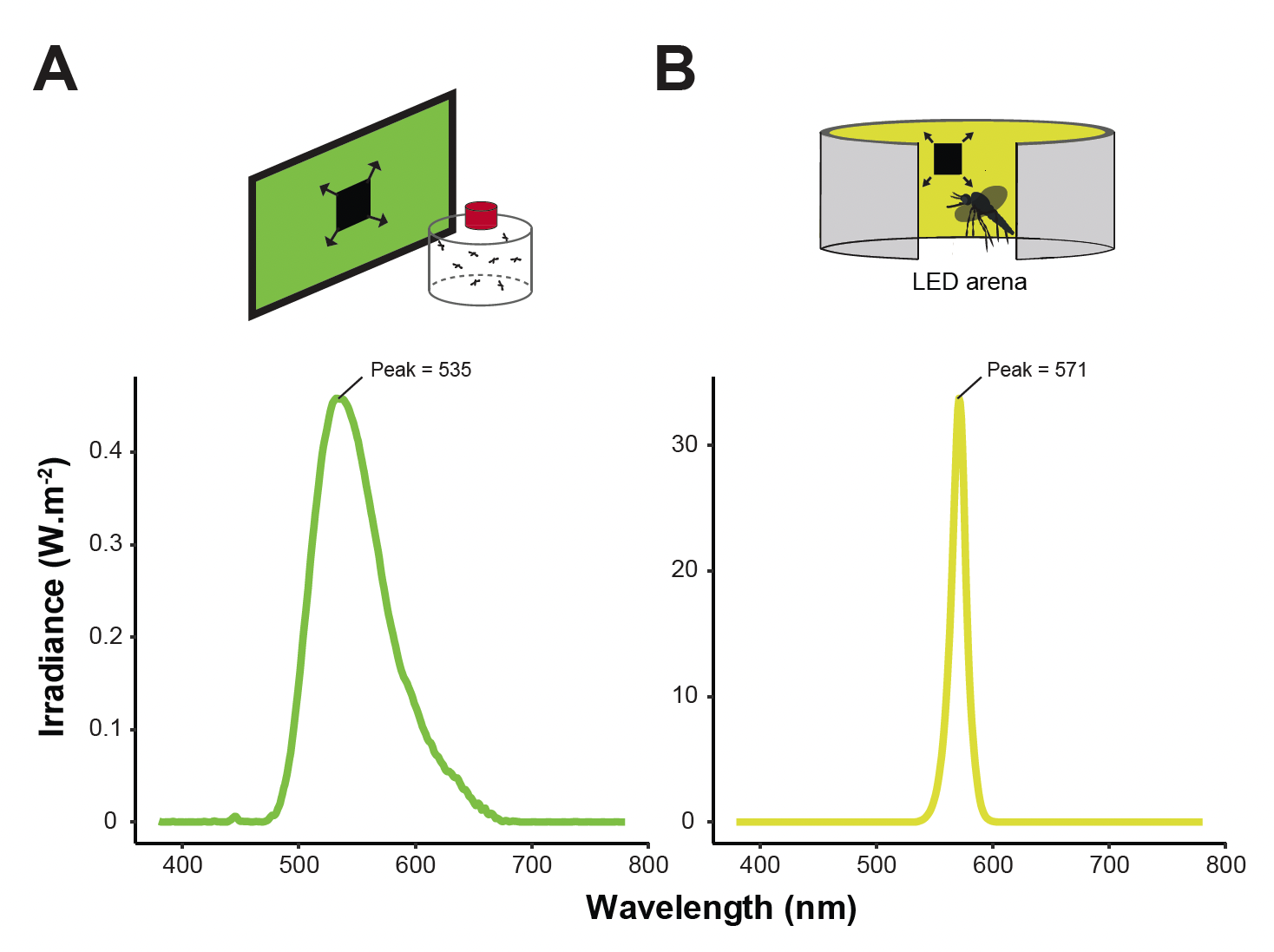
**

**Figure S1. Spectral irradiance of the visual displays.**

The spectral signature of the green backgrounds used in the feeding **(A)** and take-off **(B)** assays was determined by measuring the irradiance of displays for wavelengths between 380 and 780 nm, using a portable spectral irradiance colorimeter (OHSP350, Hopoocolor, Hangzhou China).

**
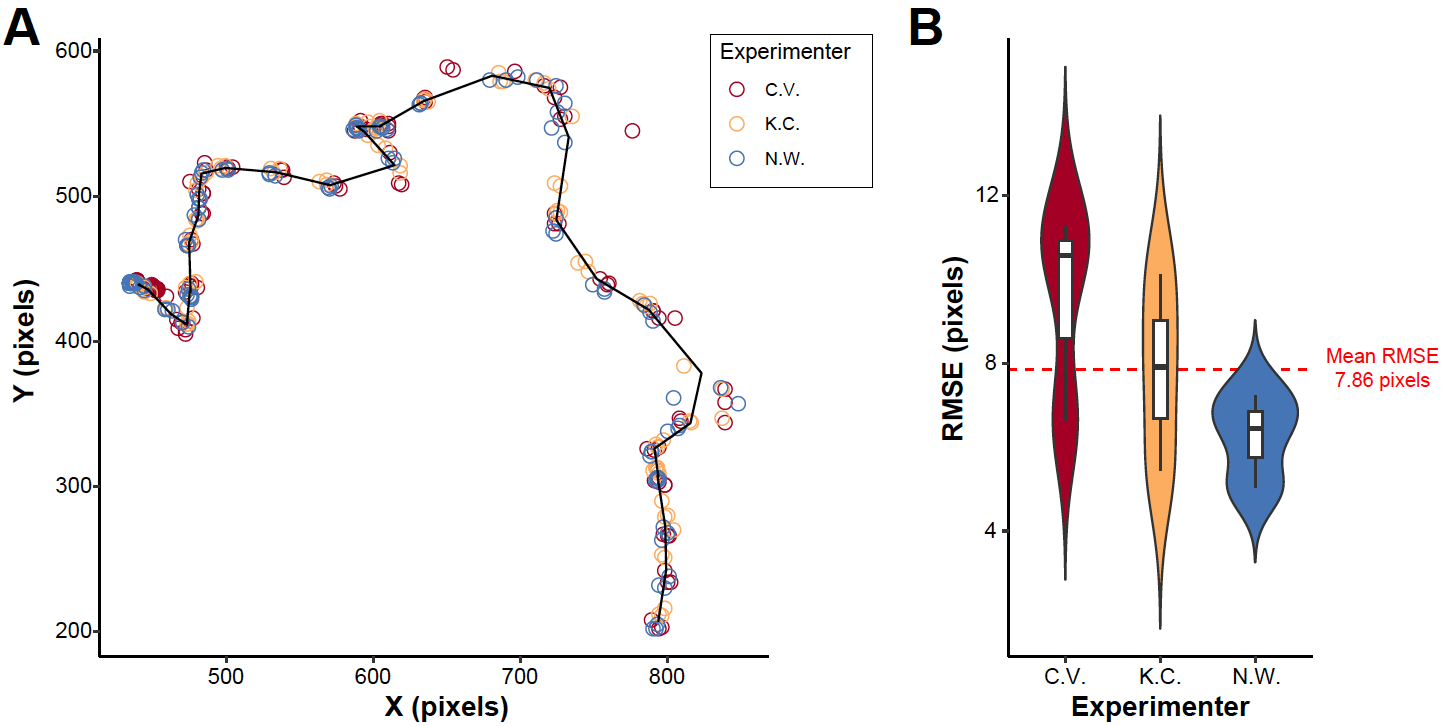
**

**Figure S2. Digitization error associated with manual tracking.**

**(A)** Tracking of a 59-frame flight sequence by 3 different experimenters on 3 consecutive days. Experimenters are identified by their initials. Data were collected in triplicates and results are color-coded for the individual experimenter. The solid black line indicates the mean trajectory calculated from the mean of the coordinates tracked by all 3 experimenters. **(B)** Digitizing error quantified as the Root Square Mean Error (RMSE), representing the average deviation from the mean trajectory, for each experimenter. The dashed red line indicates the mean RMSE.

**
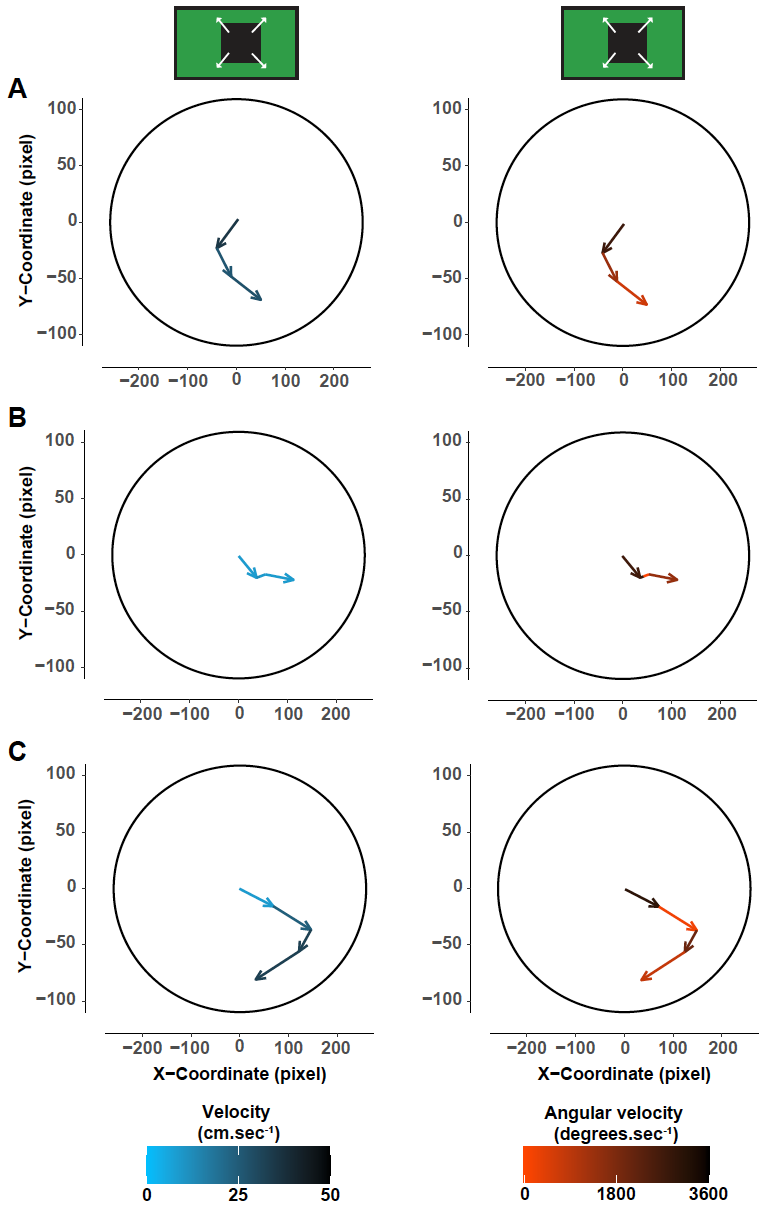
**

**Figure S3. Representative trajectories of mosquitoes’ escape (take-off) responses.**

Digitized trajectories of the escape (take-off) responses of three mosquitoes **(A-C)** to the expanding stimulus. The visualized cartesian coordinates are tracks of the respective mosquitoes’ head in the first 5 frames of the escape (take-off) response upon stimulus introduction (*i.e.,* ⅙ of a second). The trajectories are rotated to fictively re-position the stimuli introductions at the top at 0° (represented as a schematic). The trajectories are color coded as a function of the mosquitoes’ velocity (left panel, blue) and angular velocity (right panel, red).

**
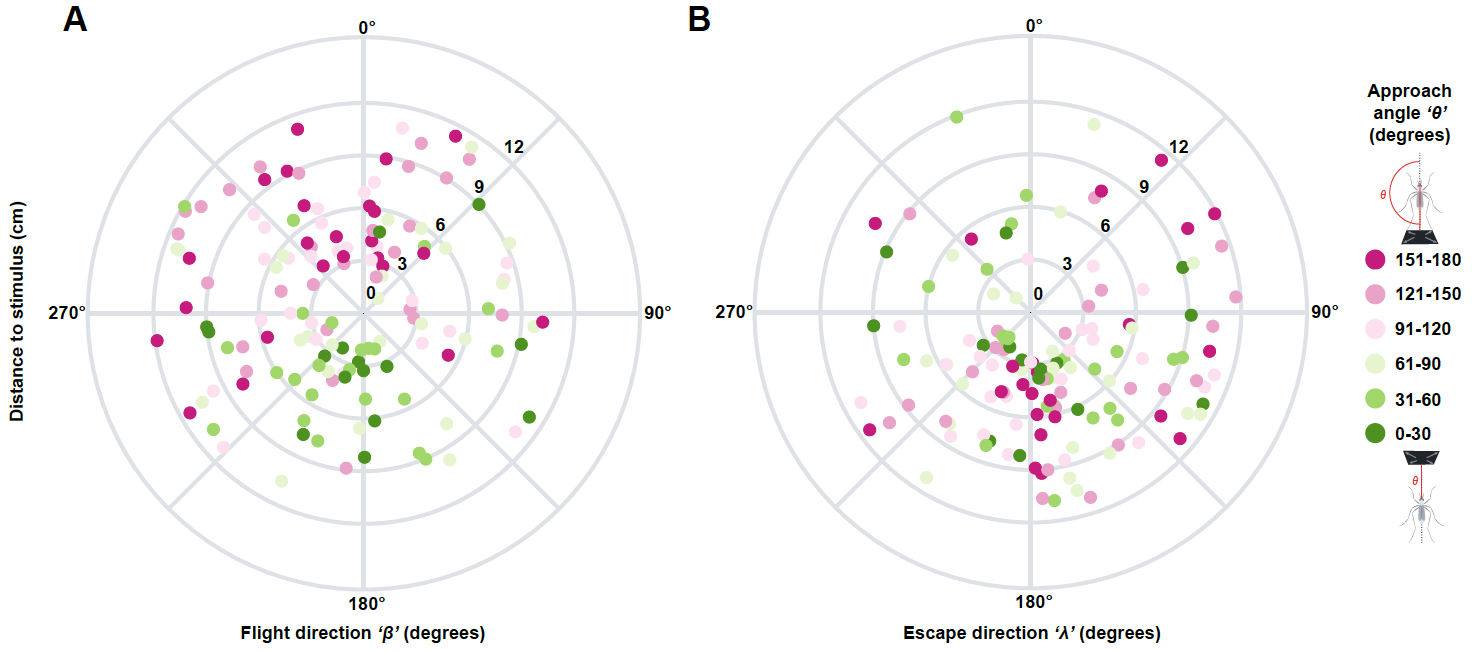
**

**Figure S4. Flight direction (**𝛽**) and escape direction (**𝜆) **as a function of mosquitoes’ distance to the stimulus**

Circle plots showing the **(A)** flight direction, 𝛽 (*i.e.,* the direction of escape relative to the mosquito’s body orientation) and **(B)** escape direction, 𝜆 (*i.e.,* the direction of escape relative to the stimulus, at 0°) within 0.17 sec of stimulus-triggered take-offs. The response variables are visualized as a function of the mosquitoes’ distance to the stimulus and color-coded as a function of the approach angle, 𝜃 where 0° is to the front of the mosquito and 180° is behind the mosquito. Each point represents one take-off (n = 142).

**
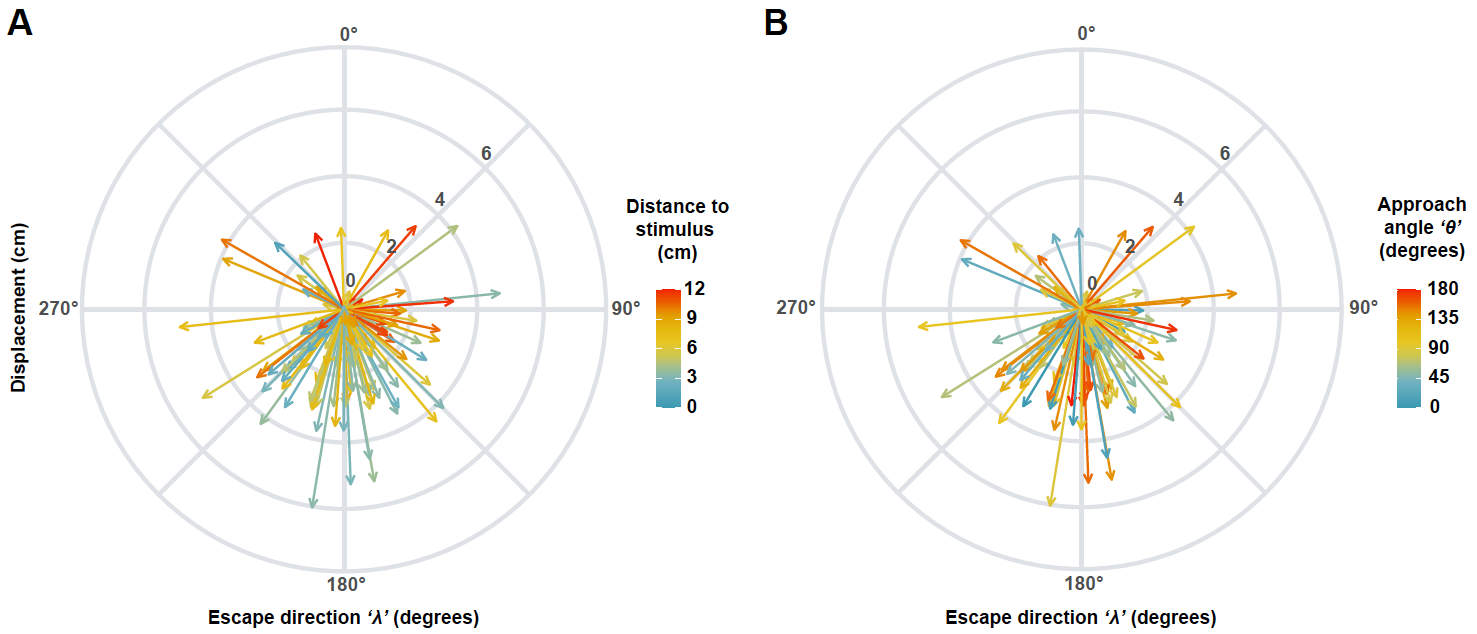
**

**Figure S5. Mosquito displacement as a function of the escape direction (𝜆) in response to the looming stimulus**

Circle plot showing the displacement of mosquitoes (*i.e.*, distance travelled on a scale ranging from 0-6 cm) within 0.17 sec of stimulus-triggered take-offs. The length of the vectors encodes for displacement, color coded as a function of the **(A)** distance to the stimulus’ point of introduction, and **(B)** approach angle, 𝜃 where 0° is to the front of the mosquito and 180° is behind the mosquito. The position of the vector around the circle encodes for the escape direction, 𝜆 (*i.e.,* the direction of escape relative to the stimulus, at 0°). Each vector represents one take-off (n = 142).

**Supplementary Table 1:** Number of mosquitoes sampled for their responses to expanding stimuli as a function of the approach angle ‘𝜃’

| **Approach angle ‘𝜃’**  **(degrees)** | **No.**  **trials** | **Lower**  **CI** | **Upper**  **CI** | **z-ratio** |
| --- | --- | --- | --- | --- |
| 0 – 10 | 22 | 14.5 | 33.4 | 14.498 |
| 10 – 20 | 23 | 15.3 | 34.6 | 15.037 |
| 20 – 30 | 21 | 13.7 | 32.2 | 13.952 |
| 30 – 40 | 20 | 12.9 | 31 | 13.397 |
| 40 – 50 | 25 | 16.9 | 37 | 16.094 |
| 50 – 60 | 22 | 14.5 | 33.4 | 14.498 |
| 60 – 70 | 24 | 16.1 | 35.8 | 15.569 |
| 70 – 80 | 27 | 18.5 | 39.4 | 17.126 |
| 80 – 90 | 23 | 15.3 | 34.6 | 15.037 |
| 90 – 100 | 26 | 17.7 | 38.2 | 16.613 |
| 100 – 110 | 28 | 19.3 | 40.6 | 17.632 |
| 110 – 120 | 32 | 22.6 | 45.3 | 19.605 |
| 210 – 130 | 37 | 26.8 | 51.1 | 21.964 |
| 130 – 140 | 35 | 25.1 | 48.7 | 21.034 |
| 140 – 150 | 55 | 42.2 | 71.6 | 29.719 |
| 150 – 160 | 47 | 35.3 | 62.6 | 26.395 |
| 160 – 170 | 42 | 31 | 56.8 | 24.223 |
| 170 – 180 | 42 | 31 | 56.8 | 24.223 |

| **Supplementary Table 2:** Number of mosquitoes sampled for their responses to expanding stimuli as a function of the mosquitoes’ distance to the expanding stimulus   \| **Distance to stimulus (cm)** \| **No.**  **trials** \| **Lower**  **CI** \| **Upper**  **CI** \| **z-ratio** \| \| --- \| --- \| --- \| --- \| --- \| \| 0 – 2 \| 13 \| 7.55 \| 22.4 \| 9.248 \| \| 2 – 3 \| 33 \| 23.46 \| 46.4 \| 20.086 \| \| 3 – 4 \| 48 \| 36.17 \| 63.7 \| 26.82 \| \| 4 – 5 \| 34 \| 24.29 \| 47.6 \| 20.562 \| \| 5 – 6 \| 55 \| 42.23 \| 71.6 \| 29.719 \| \| 6 – 7 \| 52 \| 39.62 \| 68.2 \| 28.493 \| \| 7 – 8 \| 44 \| 32.74 \| 59.1 \| 25.101 \| \| 8 – 9 \| 48 \| 36.17 \| 63.7 \| 26.82 \| \| 9 – 10 \| 68 \| 53.61 \| 86.2 \| 34.795 \| \| 10 – 11 \| 60 \| 46.59 \| 77.3 \| 31.715 \| \| 11 – 12 \| 63 \| 49.22 \| 80.6 \| 32.885 \| \| 12 – 13 \| 16 \| 9.8 \| 26.1 \| 11.09 \| \| 13 – 14 \| 17 \| 10.57 \| 27.3 \| 11.682 \| |  |
| --- | --- | --- | --- | --- | --- | --- | --- | --- | --- | --- | --- | --- | --- | --- | --- | --- | --- | --- | --- | --- | --- | --- | --- | --- | --- | --- | --- | --- | --- | --- | --- | --- | --- | --- | --- | --- | --- | --- | --- | --- | --- | --- | --- | --- | --- | --- | --- | --- | --- | --- | --- | --- | --- | --- | --- | --- | --- | --- | --- | --- | --- | --- | --- | --- | --- | --- | --- | --- | --- | --- | --- |
